# The *FTO* Gene rs9939609 Polymorphism Predicts Risk of Cardiovascular Disease: A Systematic Review and Meta-Analysis

**DOI:** 10.1371/journal.pone.0071901

**Published:** 2013-08-19

**Authors:** Chibo Liu, Sihua Mou, Chunqin Pan

**Affiliations:** 1 Department of Clinical Laboratory, Taizhou Municipal Hospital, Taizhou, Zhejiang, China; University of Oxford, United Kingdom

## Abstract

**Objective:**

Genome-wide association studies have shown that variance in the fat mass- and obesity- associated gene (*FTO*) is associated with risk of obesity in Europeans and Asians. Since obesity is associated with an increased risk of cardiovascular disease (CVD), several studies have investigated the association between variant in the *FTO* gene and CVD risk, with inconsistent results. In this study, we performed a meta-analysis to clarify the association of rs9939609 variant (or its proxies [*r* ^2^>0.90]) in the *FTO* gene with CVD risk.

**Methods:**

Published literature from PubMed and Embase was retrieved. Pooled odds ratios with 95% confidence intervals were calculated using the fixed- or random- effects model.

**Results:**

A total of 10 studies (comprising 19,153 CVD cases and 103,720 controls) were included in the meta-analysis. The results indicated that the rs9939609 variant was significantly associated with CVD risk (odds ratio = 1.18, 95% confidence interval = 1.07–1.30, *p* = 0.001 [Z test], *I* ^2^ = 80.6%, *p*<0.001 [heterogeneity]), and there was an insignificant change after adjustment for body mass index (BMI) and other conventional CVD risk factors (odds ratio = 1.16, 95% confidence interval = 1.05–1.27, *p* = 0.003 [Z test], *I* ^2^ = 75.4%, *p*<0.001 [heterogeneity]).

**Conclusions:**

The present meta-analysis confirmed the significant association of the rs9939609 variant in the *FTO* gene with CVD risk, which was independent of BMI and other conventional CVD risk factors.

## Introduction

The prevalence of obesity has greatly increased globally [1], [2]. Obesity is one of the main risk factors for the development of hypertension, metabolic syndrome, type 2 diabetes (T2D), and cardiovascular disease (CVD) [3]. Recently, a genome-wide association study has identified the fat mass- and obesity- associated gene (*FTO*) associated with higher body mass index (BMI) and risk of obesity in the Europeans [4]. Since then, the association has been replicated in other ethnic populations [5], [6]. In addition, the original publication also indicated that the *FTO* gene variant affected T2D through an association with BMI/obesity [4]. However, the subsequent studies supported the conclusion that *FTO* gene variant was associated with risk of T2D independently of BMI [5], [7]. Since obesity is a well established risk factor for CVD, it is more likely that the *FTO* gene, as the BMI/obesity related locus, might confer the risk on CVD. To date, nine papers have investigated the association between variance in *FTO* gene and CVD risk [8]–[16]. However, the results have been inconsistent, which might be due to the differences in statistical power for each included study (the sample size in each study varied greatly), recruitment of study population, genetic and environmental background.

Meta-analysis is a useful method to overcome the disadvantages of individual studies, thereby increasing the statistical power and the precision of effect estimates. In this study, we performed a meta-analysis to clarify the association between *FTO* gene rs9939609 variant (or its proxies [*r* ^2^>0.90]) [5] and the risk of CVD.

## Methods

### Literature and Search Strategy

We searched literature databases, including PubMed and Embase. The search strategy was to identify all possible studies involving the following key words: (fat-mass and obesity-associated gene or *FTO*) and (polymorphism or variant or variation or genotype) and (acute coronary syndromes or myocardial infarction or coronary artery disease or coronary heart disease or ischemic heart disease or cardiovascular disease or stroke). The publication language was restricted to English. The reference lists of retrieved articles were hand-searched. The literature search was updated on 31 December 2012.

### Inclusion Criteria and Data Extraction

The studies were included in the meta-analysis only if they met all the following inclusion criteria: (1) evaluation of the association of *FTO* rs9939609 polymorphism (or its proxies [*r* ^2^>0.90]) with risk of CVD; (2) use of a case–control or cohort design; and (3) provision of an odds ratio (OR) with 95% confidence interval (CI) under an additive model with or without adjustment for BMI and other conventional CVD risk factors (e.g., age, sex, alcohol use, caloric intake, saturated fat and fiber intake, smoking, and obesity). The following information was extracted from each study: (1) name of the first author; (2) year of publication; (3) country of origin; (4) ethnicity of the studied population; (5) number of cases and controls; (6) endpoint; (7) frequency of men and the mean ages; (8) mean BMI; and (9) studied SNP. Two authors independently reviewed the articles for compliance with the inclusion/exclusion criteria, resolved disagreements and reached a consistent decision. All participants of the included studies provided informed consent and the studies were approved by the ethics committees of the participating institutions.

### Statistical Analysis

The association of *FTO* polymorphism with CVD was estimated by calculating the pooled OR and 95% CI. The significance of the OR was determined by the *Z* test (*p*<0.05 was considered statistically significant). Cochrane’s *Q* test was performed to test the between-study heterogeneity [17], [18]. *I* ^2^ represents the range for the existence or no of heterogeneity. Usually, *I* ^2^>50% represents the existence of heterogeneity. A random-effects (DerSimonian–Laird [17]) or fixed-effects (Mantel–Haenszel [18]) model was used to calculate the pooled OR in the presence (*p*≤0.10) or absence (*p*>0.10) of heterogeneity, respectively. To evaluate the stability of the results, we performed a sensitivity analysis by removing one study at a time. Publication bias was assessed by Begg’s test [19] (*p*<0.05 was considered statistically significant). Data were analysed using STATA version 11.0 (StataCorp LP, College Station, TX, USA).

## Results

### Characteristics of the Studies

**Figure 1:**
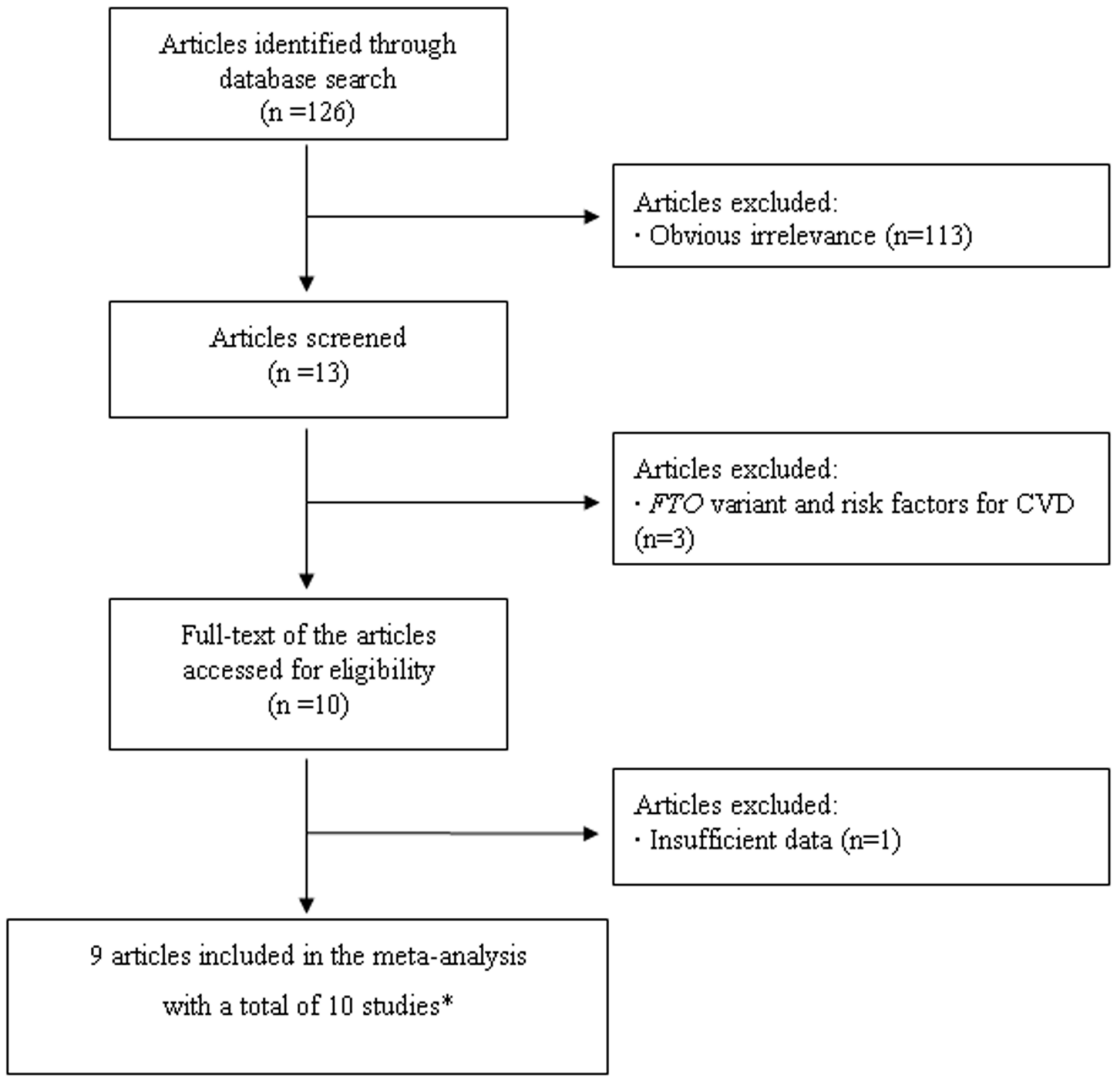
Flow chart of meta-analysis for exclusion/inclusion of individual articles (or studies). ^*^ One article (Lappalainen et al, 2011) contained two studies.

A flow chart describing the process of inclusion/exclusion of studies is presented in Fig. 1. The literature search identified a total of 126 potentially relevant articles. Of these, 113 were excluded after reading the title or abstract because of obvious irrelevance. In addition, three articles were excluded as they investigated the association between the *FTO* polymorphism and risk factors for CVD, e.g., obesity, hypertension, diabetes and insulin resistance [20]–[22]. Therefore, 10 articles met the primary inclusion criteria, of which one article was excluded because it did not provide the sufficient data for calculation of an OR with 95% CI [23]. In addition, the two studies included in the paper by Lappalainen et al. [12] were considered as separate studies in the following data analysis. The variant rs9939609 is known to be in high linkage disequilibrium with proxies including rs8050136, rs17817449 and rs9937053 (All *r* ^2^>0.90). A total of 10 studies (comprising 19,153 CVD cases and 103,720 controls) for rs9939609 polymorphism (or its proxies) were included in the meta-analysis [8]–[16]. All studies were conducted in the European populations. The genotype frequency in controls was in Hardy–Weinberg equilibrium for all included studies (*p*>0.05). All studies (except for one study in the paper by Lappalainen et al. [12]) provided the crude and adjusted (adjusted for BMI and other conventional CVD risk factors calculated by multiple logistic regression model for each study) ORs with 95% CIs, and the majority provided OR with 95% CI under an additive genetic model, hence we calculated the summary estimate under this model only. All included studies were conducted in European population. The characteristics of the included studies are listed in Table 1.

**Table 1:** Characteristics of individual studies included in the meta-analysis.

Authors (ref)	Country	Ethnicity	Sample size		Endpoint	Sex (% men)	Age (Mean [SD], years)	BMI (Mean [SD], kg/m^2^)	*FTO* SNP (risk/non-risk allele)	Genotyping method	Statisticalpower a
			Cases	Controls							
Doney, 2009 (8)	UK	European	324	3777	MI	All: 45.7	All: 63.7 (11.7)	All: 30.7 (5.9)	rs9939609(A/T)	TAQMAN, KASPAR	0.886
Ahmad, 2010 (9)	USA	European	555	18271	CVD	All were women	All: 52.0 (48.0–59.0)	All: 24.9 (22.5–28.3)	rs8050136(A/C)	Illumina	0.989
He, 2010 (10)	USA	European	425	901	CVD	All were women	Cases: 60 (6)Controls: 57 (7)	Cases: 30.1(6.0)Controls: 30.1(7.0)	rs9939609(A/T)	OpenArray™	0.875
Hubacek, 2010 (11)	Czech	European	1092	1191	ACS	All were men	Cases: 55.2 (7.5)Controls: 49.0 (10.8)	Cases: 28.5 (4.3)Controls: 28.2 (4.0)	rs17817449(G/T)	PCR–RFLP	0.992
Lappalainen, 2011 (12)	Finland	European	250	240	CVD	All: 49.8	All: 55.3 (7.0)	All: 31.3 (4.6)	rs9939609(A/T)	TaqMan	0.524
Lappalainen, 2011 (12)	Finland	European	851	5363	CVD	All were men	All: 58.7 (6.4)	All: 27.3 (4.2)	rs9939609(A/T)	TaqMan	0.998
Winter, 2011 (13)	Germany	European	379	379	Stroke	Cases: 62.8Controls: 62.8	Cases: 67 (11)Controls: 65 (9)	Cases: 28 (4)Controls: 27 (4)	rs9937053(A/T)	TaqMan	0.710
Berzuini, 2012 (14)	Italy	European	1838	1838	MI	NA	NA	NA	rs9939609(A/T)	MassARRAY	0.999
Borglykke, 2012 (15)	Denmark	European	2383	7189	CVD	MONICA 1: 50.9Inter99: 48.1	MONICA 1: 45.0 (7.3)Inter99: 45.9 (7.9)	MONICA 1: 24.6 (3.9)Inter99: 26.2 (4.6)	rs9939609(A/T)	KASPar	0.999
Nordestgaard, 2012 (16)	Denmark	European	11056	64571	IHD	CGPS: 44.4CCHS: 44.4CIHDS: 57.0	CGPS: 57 (47–67)CCHS: 60 (47–70)CIHDS: 60 (51–69)	CGPS: 25.6 (23.2–28.5)CCHS: 23.9 (21.8–26.7)CIHDS:25.5 (23.3–28.1)	rs9939609(A/T)	ABI PRISM 7900HT	0.999

MI, myocardial infarction; CVD, cardiovascular disease; ACS, acute coronary syndrome; IHD, ischemic heart disease; NA, not available; MONICA, the study focus on multinational monitoring of trends and determinants in cardiovascular disease; Inter99, the study focus on effect on IHD incidence of individually tailored non-pharmacological intervention on lifestyle using a newly developed computer-based health educational tool; CGPS, Copenhagen General Population Study; CCHS, Copenhagen City Heart Study; CIHDS, Copenhagen Ischemic Heart Disease Study.

a The power calculation was performed using Quanto software http://hydra.usc.edu/gxe/.

### Meta-analysis Results

**Figure 2:**
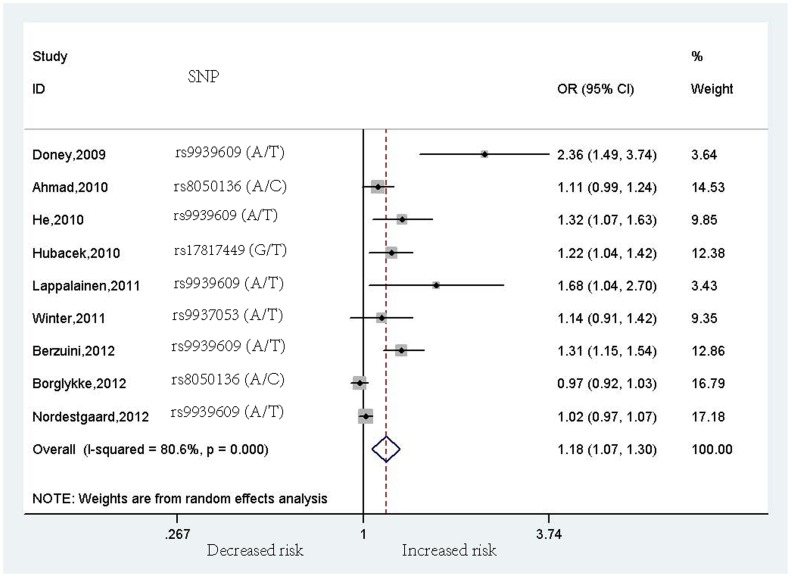
Meta-analysis of the association between rs9939609 (or its proxies) polymorphism in the *FTO* gene and cardiovascular disease risk. OR is reported to increased CVD risk; weights are calculated from the inverse of their variance.

**Figure 3:**
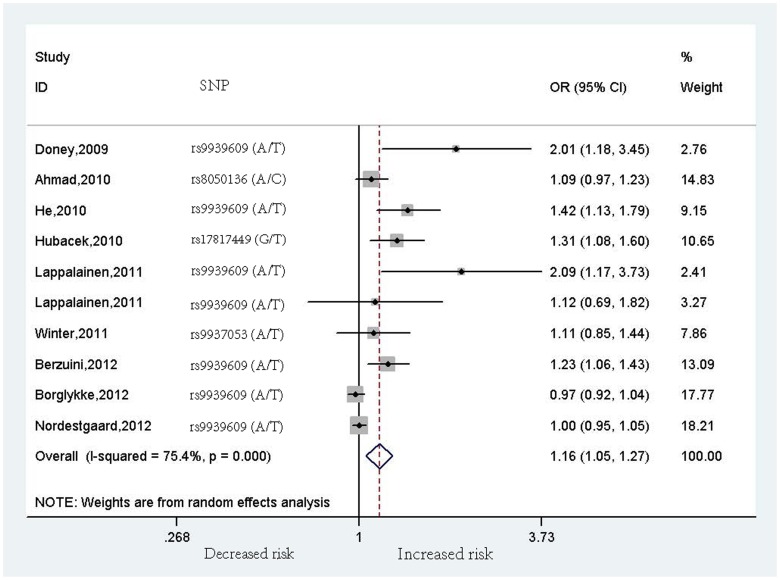
Meta-analysis of the association between rs9939609 (or its proxies) polymorphism in the *FTO* gene and cardiovascular disease risk after adjustment for BMI and other conventional CVD risk factors. OR is reported to increased CVD risk; weights are calculated from the inverse of their variance.

The results indicated a significant association of the rs9939609 polymorphism in the *FTO* gene with the risk of CVD (OR = 1.18, 95% CI = 1.07–1.30, *p* = 0.001 [Z test], *I* ^2^ = 80.6%, *p*<0.001 [heterogeneity], Fig. 2), which did not substantially change after adjustment for BMI and other conventional CVD risk factors (OR = 1.16, 95% CI = 1.05–1.27, *p* = 0.003 [Z test], *I* ^2^ = 75.4%, *p*<0.001 [heterogeneity], Fig. 3).

### Source of Heterogeneity

Meta-regress was conducted to examine the source of heterogeneity. Publication year, country, design, number of cases and controls were considered as independent variables. However, these variables can’t explain the source of heterogeneity (all *p*>0.05). Galbraith figure was drawn to identify outliers of the source of heterogeneity. In the model unadjusted for BMI, four studies [8], [10], [14], [15] were the outliers (Figure S1). In the model adjusted for BMI, five studies [8], [10], [11], [12], [14] were the outliers (Figure S2).

### Sensitivity Analysis

Sensitivity analysis was performed by excluding one study at a time. The results confirmed the significant association between the rs9939609 polymorphism in the *FTO* gene and the risk of CVD, with ORs and 95% CIs ranging from 1.13 (1.04, 1.24) to 1.23 (1.09, 1.39) (Table S1), and from 1.12 (1.02, 1.23) to 1.22 (1.07, 1.39) after adjustment for BMI and other conventional CVD risk factors (Table S2).

### Publication Bias

No publication bias was observed for the association between rs9939609 polymorphism and CVD risk without (*p* = 0.118, Figure S3) or with (*p* = 0.152, Figure S4) adjustment for BMI other conventional CVD risk factors.

## Discussion

To our knowledge, this is the first meta-analysis investigating the association between the *FTO* gene variant and CVD risk. Our meta-analysis with 19,383 CVD cases and 103,490 controls indicated that the *FTO* gene rs9939609 variant was significantly associated with an increased risk of CVD and the association did not substantially alter even after adjustment for BMI and other conventional CVD risk factors.

In 2007, Frayling et al. [4] initially reported that the *FTO* gene rs9939609 variant was significantly associated with T2D in Europeans, which was completely explained by the effect of the *FTO* gene variant on BMI. However, the recent two meta-analyses suggested that the *FTO* gene variant influenced the risk of T2D independently of BMI in both Europeans and East Asians [5], [7]. Regarding for the association between *FTO* gene and CVD risk, Doney et al. [8] firstly demonstrated that the A allele of rs9939609 in the *FTO* gene increased the risk of myocardial infarction in 4,897 patients with T2D in the prospective study, which was independently of BMI, glycohemoglobin, mean arterial pressure, HDL-C, triglycerides, and total cholesterol. However, the subsequent eight papers revealed conflicting conclusions [9]–[16]. Pooling all the studies together, we found the significant association between the *FTO* gene variant and CVD risk independent of BMI and other conventional CVD risk factors.

The mechanism underlying the association of the *FTO* variant with CVD risk remains unclear. As is known, FTO protein is highly expressed in the central nervous system, which regulates energy metabolism [24]. Indeed, the *FTO* gene variant is found to influence energy-dense food intake instead of regulation of energy expenditure [25]. In addition, the *FTO* variant is associated with diabetes-related metabolic traits (including higher fasting insulin, glucose and triglycerides, and lower HDL cholesterol), although the association disappeared after adjustment for BMI [26]. Other studies have indicated that *FTO* variant is associated with increased risk for hypertension through the regulation of sympathetic modulation of vasomotor tone [27]. Notably, Hubacek et al. [11] believed that the *FTO* variant could increase the risk of CVD through another mechanism, namely through its possible effect on DNA methylation. In other words, the *FTO* gene variant could interact with an unhealthy lifestyle (such as high fat diet and lack of physical activity), and affect the epigenetic status [11] and ultimately contribute to the development of CVD.

Our study is subject to several limitations. First, the individual studies are not homogeneous in the subject cases that might have an effect in the meta-analysis (Table 1). Only five studies provided the specific endpoint (two were on myocardial infarction, one was on acute coronary syndrome, one was on stroke and one was on ischemic heart disease) and the remaining five studies indicated CVD only. Thus, we were unable to perform the subgroup analysis to observe the effect of different endpoints on the association. Second, we only calculated the pooled OR under an additive model since most studies provided OR with 95%CI under this model only. In other word, the insufficient data under dominant and recessive models impeded us for further analysis. Third, the genotyping methods are different among the included studies, which may influence the association between *FTO* gene variant and CVD risk.

In conclusion, our meta-analysis confirmed that the *FTO* gene rs9939609 variant is significantly associated with an increased risk of CVD in the Europeans and the genetic effect is not mediated by the changes in BMI and other conventional CVD risk factors. The observed association remains to be replicated in the non-European populations. Further studies should be conducted to investigate the mechanism underlying the link between *FTO* gene and CVD risk.

## Supporting Information

Figure S1 **Galbraith figure for identification outliers of the source of heterogeneity in the model unadjusted for BMI.**
(TIF)

Figure S2 **Galbraith figure for identification outliers of the source of heterogeneity in the model adjusted for BMI.**
(TIF)

Figure S3 **Funnel plot for the association between rs9939609 polymorphism and CVD risk without adjustment for BMI.**
(TIF)

Figure S4 **Funnel plot for the association between rs9939609 polymorphism and CVD risk with adjustment for BMI.**
(TIF)

Table S1 (DOC)

Table S2 (DOC)
