# Supplementary material for: The FTO Gene rs9939609 Polymorphism Predicts Risk of Cardiovascular Disease: A Systematic Review and Meta-Analysis
Source: PLoS One. 2013 Aug 19;8(8):e71901. doi: 10.1371/journal.pone.0071901 (PMC3747067; doi:10.1371/journal.pone.0071901)
Supplement: Table S2 — (DOC) [file pone.0071901.s006.doc]

Table S2 Sensitivity analysis of the association between rs9939609 (or its proxies) polymorphism in the *FTO* gene and cardiovascular disease risk after adjustment for BMI and other conventional CVD risk factors

| Study omitted | OR | 95% CI | |
| --- | --- | --- | --- |
| Doney,2009 | 1.13 | 1.03 | 1.24 |
| Ahmad,2010 | 1.18 | 1.05 | 1.31 |
| He,2010 | 1.12 | 1.02 | 1.23 |
| Hubacek,2010 | 1.13 | 1.03 | 1.25 |
| Lappalainen,2011 | 1.13 | 1.03 | 1.24 |
| Lappalainen,2011 | 1.16 | 1.05 | 1.28 |
| Winter,2011 | 1.16 | 1.05 | 1.29 |
| Berzuini,2012 | 1.14 | 1.03 | 1.26 |
| Borglykke,2012 | 1.22 | 1.08 | 1.38 |
| Nordestgaard,2012 | 1.22 | 1.07 | 1.39 |
